# Gold vs. Silver Colloidal Nanoparticle Films for Optimized SERS Detection of Propranolol and Electrochemical-SERS Analyses

**DOI:** 10.3390/bios13050530

**Published:** 2023-05-09

**Authors:** Cristina M. Muntean, Denisa Cuibus, Sanda Boca, Alexandra Falamas, Nicoleta Tosa, Ioana Andreea Brezeştean, Attila Bende, Lucian Barbu-Tudoran, Rebeca Moldovan, Ede Bodoki, Cosmin Farcǎu

**Affiliations:** 1 National Institute for Research and Development of Isotopic and Molecular Technologies, 67-103 Donat Str., 400293 Cluj-Napoca, Romania; 2 Institute for Interdisciplinary Research in Bio-Nano-Sciences, Babeş-Bolyai University, 42 T. Laurian Str., 400271 Cluj-Napoca, Romania; 3 Analytical Chemistry Department, Faculty of Pharmacy, “Iuliu Hațieganu” University of Medicine and Pharmacy, 4, Louis Pasteur, 400349 Cluj-Napoca, Romania

**Keywords:** β-blocker propranolol, surface-enhanced Raman spectroscopy, self-assembled nanoparticle films, DFT calculations

## Abstract

The increasing pollution of surface and groundwater bodies by pharmaceuticals is a general environmental problem requiring routine monitoring. Conventional analytical techniques used to quantify traces of pharmaceuticals are relatively expensive and generally demand long analysis times, associated with difficulties in performing field analyses. Propranolol, a widely used β-blocker, is representative of an emerging class of pharmaceutical pollutants with a noticeable presence in the aquatic environment. In this context, we focused on developing an innovative, highly accessible analytical platform based on self-assembled metal colloidal nanoparticle films for the fast and sensitive detection of propranolol based on Surface Enhanced Raman Spectroscopy (SERS). The ideal nature of the metal used as the active SERS substrate was investigated by comparing silver and gold self-assembled colloidal nanoparticle films, and the improved enhancement observed on the gold substrate was discussed and supported by Density Functional Theory calculations, optical spectra analyses, and Finite-Difference Time-Domain simulations. Next, direct detection of propranolol at low concentrations was demonstrated, reaching the ppb regime. Finally, we showed that the self-assembled gold nanoparticle films could be successfully used as working electrodes in electrochemical-SERS analyses, opening the possibility of implementing them in a wide array of analytical applications and fundamental studies. This study reports for the first time a direct comparison between gold and silver nanoparticle films and, thus, contributes to a more rational design of nanoparticle-based SERS substrates for sensing applications.

## 1. Introduction

The pollution of various sources of freshwater bodies and wastewater by pharmaceuticals is a general environmental problem that requires routine monitoring of pollutants. Conventional, separation-based, high-performance analytical methods that are used to quantify low levels of pharmaceuticals are relatively expensive and generally demand long analysis times or the use of toxic reagents, and are also associated with difficulties in performing on-field investigations. The sample preparation phase includes separation and/or pre-concentration steps, which can be problematic for a range of polar-type pharmaceuticals [1,2]. Moreover, even with the recent improvements in some of the used methods, such as solid-phase microextraction, lower detection precisions have been obtained, pointing out the need for further optimization [3]. Additionally, issues still remain with the fast and accurate simultaneous detection of a large number of pharmaceuticals in complex matrices, as well as with decreasing the limit of detection (LoD). Finally, the need for enhancing the monitoring approaches points toward the development of analytical methods that require less expensive and sophisticated instruments that could, if needed, be used for on-field investigations, as well.Raman spectroscopy and surface-enhanced Raman spectroscopy (SERS) may represent promising analytical alternatives as very powerful spectroscopic tools, allowing sensitive detection of low-concentration analytes, including pharmaceutical compounds [4,5]. However, experimental Raman/SERS spectra of various pharmaceutical molecules are not yet completely understood, and concerns related to the reproducibility and ruggedness of such analysis have also been raised [6].

Along with non-steroidal anti-inflammatory drugs (NSAIDs), lipid regulators, and antibiotics, the representatives of beta-adrenergic receptor antagonists (β-blockers) are the most often prescribed drugs. However, they are commonly encountered as one of the emerging classes of pharmaceutical pollutants in surface and groundwaters across Europe. Various regulating bodies around the world have raised awareness of the growing risks of pharmaceutical product residues entering the environment and, in particular, water bodies during their manufacture, use, and disposal [7]. Propranolol (PRNL), as the first clinically important β-blocker, is widely used in the treatment of various cardiovascular diseases, such as hypertension, angina pectoris, and tachycardia, and also for the prevention or ailment of other conditions, such as migraine, anxiety, essential tremors, and hyperthyroidism [8].

Different highly sensitive and (stereo) selective SERS-based methods for the detection of trace amounts of propranolol from various matrices have already been reported in the literature. For example, a sandwich-type molecular imprinted polymer-based nanostructure consisting of a composite graphene oxide/molecular imprinted polymer and silver nanoparticles was proved efficient for the ultrasensitive SERS detection of propranolol [9]. Aiming to improve the recorded SERS signal and its reproducibility, the enhancement of SERS detection for propranolol with multiobjective evolutionary optimization was also investigated [10]. Additionally, metal nanoparticle-mediated selective recognition of propranolol enantiomers through native cyclodextrin complexation using SERS has been described [11]. Furthermore, Farcaş et al. reported the results of a systematic Raman, SERS, and Density Functional Theory (DFT) study on four beta-blocking molecules, including propranolol [12]. Based on DFT calculations, the FT-IR, Raman, and SERS spectra of propranolol enantiomers were assigned. The adsorption geometry of both enantiomers onto the silver surface was predicted using the calculated molecular electrostatic potential in association with data obtained from SERS [13]. Another study reported SERS and conventional Raman spectra of four β-blocker drugs, including propranolol. The authors used silver colloidal dispersions prepared with simple sodium borohydride reduction of silver nitrate as SERS substrates for which they obtained spectral fingerprints at trace levels [14]. Most of the studies cited above were performed on colloidal suspensions of metal nanoparticles. Due to several reasons, such as reproducibility, stability, as well as a more predictable signal enhancement, solid SERS substrates would be preferable. Moreover, one of the aspects still not clarified is whether gold or silver would be most effective as a SERS substrate for the particular detection of propranolol.

Robust and versatile strategies for the development of functional nanostructured materials often focus on assemblies of metallic nanoparticles. Research interest in such assemblies arised due to their potential applications in the fields of photonics and sensing [15]. Regarding the design of SERS active substrates, various strategies were proposed, such as chemical synthesis, template-assisted methods, or deterministic (lithographic) patterning [16]. Particularly, metal colloids have been widely used due to their ease of preparation and high SERS activities, allowing detection at the level of single-molecule [17]. Electron beam lithography (EBL), on the other hand, has the advantage of excellent applicability to patterning both periodic and aperiodic arbitrary shapes. Nevertheless, the technique has been explored in the area of SERS far less extensively because of the limited availability of EBL tools, as well as the high processing cost compared to chemical synthesis [16]. A good compromise could be the development of solid substrates using colloids as building blocks. Indeed, the assembly of metal nanoparticles to produce SERS-active substrates demonstrated its usefulness in several cases [18,19]. Convective self-assembly (CSA) is a simple manufacturing technique for obtaining particle-based films by their evaporative-induced assembly from colloidal suspensions on solid substrates [20]. This technique was mostly applied to assemble microspheres (hundreds of nm in size) and has been rarely employed for nanoparticles [21]. Despite the fact that the high-density particle packing favors strong electromagnetic fields and makes these films appropriate for SERS, such metallic nanoparticle films made using CSA have rarely been used as SERS substrates [22].

The development of platforms for electrochemical (EC)-SERS measurements is also of particular interest, which stems from the numerous advantages posed by the coupling of the two analytical techniques, such as higher sensitivity and selectivity, efficient adsorption with maximum surface coverage, reusability, and improved reproducibility [6]. Therefore, the successful development of EC-SERS sensors for the trace detection of PRNL could lead to real-life applications, especially in biomedical and environmental analysis.

In this work, we present a SERS study of propranolol adsorbed on two types of noble metal nanoparticle films: silver and gold, the two most efficient plasmonic metals for SERS. The nanoparticle films are directly obtained by CSA from colloidal suspensions on solid substrates. Three main objectives are targeted: (i) to determine which of these two plasmonic metals is the most efficient for SERS detection of PRNL and understand why; (ii) to assess the possibility of implementing such self-assembled colloidal nanoparticle films for SERS-based detection of PRNL at low concentrations; and (iii) to explore whether the CSA SERS substrates could be used as a dual, working electrode/substrate for EC-SERS measurements.

Ag- and Au-based SERS substrates are among the most common metals used in SERS experiments. Despite more recent research focusing on other types of metals and metal oxides as SERS substrates, Au and Ag still offer the highest enhancement factors (EFs) [23]. Generally, Ag nanoparticles (NPs) presented stronger plasmonic properties and higher EFs than Au NPs [24]; however, they show lower chemical stability and biocompatibility. Additionally, the same substrate can provide different EFs and limits of detection depending on the analyte and the measurement conditions. Therefore, it is important to determine the best type of metal to be used as the SERS substrate for a specific analyte. Our proposed strategy requires no sample preparation steps to detect trace levels of PRNL in water samples compared to conventionally used analysis methods. Moreover, the SERS signal can be detected with simple, accessible Raman instruments that can be used when needed for field detection. DFT calculations investigating the adsorption energy of the molecule near metal surfaces, as well as the SERS experiments, were analyzed to better understand the affinity of PRNL toward the metal surface. Optical characterizations and electromagnetic simulations were employed to assess the plasmonic response of the nanoparticle films. The detection of PRNL down to 10^−7^ M is shown on gold nanoparticle films without any specific surface functionalization scheme. Finally, Au NP films were assembled on top of flat Au electrodes and successfully used for the first time for the analysis of PRNL using EC-SERS. The direct comparison between gold and silver nanoparticle films yields information for a more rational design of nanoparticle-based SERS substrates for sensing applications. The self-assembled gold nanoparticle films are also promising as working electrodes in electrochemical-SERS analyses, opening the possibility of their implementation in a wide array of analytical applications and fundamental studies.

## 2. Materials and Methods

### 2.1. Colloidal Nanoparticle Syntheses

Ag colloidal suspensions were prepared according to the classical Lee and Meisel chemical reduction synthesis procedure [25]. A total of 100 mL of aqueous solution containing 17 mg of AgNO_3_ salt was heated to the boiling point (100 °C), and then 2 mL of 1% trisodium citrate solution was added dropwise under constant stirring. The suspension was further heated for 45 min at 100 °C and then allowed to cool to ambient temperature. The freshly prepared colloidal Ag NPs solution showed a milky grey color. Au nanoparticles (Au NPs) of ~50 nm were synthesized by the aqueous reduction of HAuCl_4_ with trisodium citrate according to an adapted version of the Turkevich–Frens protocol. Briefly, 50 mL of 2.5 × 10^−4^ M HAuCl_4_·3H_2_O was heated until boiling. A solution of trisodium citrate (C_6_H_5_Na_3_O_7_·2H_2_O, 1% *w*/*v*) was quickly added, and the mixture was kept under stirring until it changed color from yellow to pinky-red. Then, the colloid was removed from the heat, stirred for another 15 min for the complete reduction of gold ions, and stored at 4 °C until further use.

### 2.2. Convective Self-Assembly of Nanoparticle Films

The synthesized colloidal nanoparticles were then deposited into films on solid support by CSA. The substrates were cleaned by rinsing in ethyl alcohol and isopropyl alcohol. After washing, they were dried with nitrogen, and a UV-ozone treatment was applied for 20 min to hydrophilize the surface. CSA was performed using custom-made equipment. An amount of suspension (~10 μL) was injected between the substrate and a deposition plate positioned near the substrate, angled at about 25°. All samples were prepared in ambient conditions. CSA was also applied on top of flat gold electrodes fabricated using magnetron sputtering through stencil masks. In this case, in order to promote a stronger adhesion of the nanoparticle films to the gold electrodes, these electrodes were functionalized with 1,6-hexanedithiol 96% purity (immersion in 5 × 10^−4^ M ethanol solutions, for 20 h) prior to NP deposition.

### 2.3. Characterization

Scanning Electron Microscopy (SEM) images were obtained using a Hitachi SU8230 system operating at accelerating voltages up to 30 kV and magnifications up to 150,000×. Transmission Electron Microscopy (TEM) images were recorded using a Hitachi HD-2700, equipped with a cold field emission gun, working at 200 kV acceleration voltage. The UV-VIS-NIR reflectance measurements were performed with an HR2000+ optical fiber spectrometer working in the 200–1100 nm spectral range, with an optical resolution of 6.8 nm. The samples were placed on a Stage-RTL-T reflection stage and illuminated with a DH-2000-BAL balanced deuterium tungsten halogen source emitting in the 200–2500 nm domain. The reflected light was collected from the samples by a reflection/backscatter probe QR230-XSR having a 6-around-1 fiber bundle design, with the 6-fiber leg connecting to the light source and the single-fiber leg connecting to the spectrometer. All optical fibers had a core diameter size of 230 μm. The STAN-SSH high-reflectivity specular reflectance standard was used as a reference, and the subsequent non-unity correction has been applied. All components of the reflectivity setup are from Ocean Optics, and the spectra were recorded using the Spectra Suite 2.0.8 software.

### 2.4. SERS Measurements and Analysis

Propranolol hydrochloride solutions of various concentrations (from 10^−7^ M to 10^−2^ M) were prepared by dissolving the solid standard in methanol. In order to compare different substrates, 3 μL drops of alcoholic 10^−4^ M solution were deposited on each sample. Similarly, in order to test the concentration-dependent SERS signal, the same volume of lower PRNL concentrations (10^−5^ M, 10^−6^ M, and 10^−7^ M) were also deposited on the Au NP films. All samples were allowed to dry prior to SERS analysis. SERS measurements were performed on a Witec Alpha 300R system using a 785 nm excitation laser. A 50× (NA = 0.75) objective was used for recording the spectra. The laser power used and integration time/spectrum were 0.85 mW and 30 s (for Au NP–Ag NP comparison), as well as 0.65 mW and 60 s (for the concentration dependence study).

Principal Components Analysis (PCA) of SERS spectra was performed using the Statistics and Machine Learning Toolbox in Matlab R2016b. 

### 2.5. EC-SERS Measurements

For electrochemical–SERS experiments, a 785 nm AvaSpec-Hero fiber-optic based Raman spectrometer (Avantes B.V., Apeldoorn, The Netherlands) integrated with a Nikon Eclipse Ci-L microscope (Nikon Europe B.V., Amsterdam, The Netherlands) and a portable Sensit Smart potentiostat (Palmsens, Houten, The Netherlands) were used. A three-electrode electrochemical cell was employed, where the SERS substrate represented the working electrode, while the reference and auxiliary electrodes were represented by a leakless Ag/AgCl and Pt/Ti wire (eDAQ, Denistone, NSW, Australia), respectively. As a reference for EC-SERS measurements and for the assessment of the optimal polarization potential in the case of PRNL, electrochemically-roughened screen-printed gold electrodes were used. The working electrode was positioned under the microscope, and the other two electrodes were fixed accordingly with the aid of extension clamps. A total of 250 µL of 10^−5^ M aqueous PRNL solution in 0.05 M phosphate buffer saline (PBS), pH 7 was dispensed in the electrochemical cell, the liquid sample being in good contact with the electrodes under the confinement of an O-ring. The recording of EC-SERS spectra was performed under a 20× objective with 3.05 mW laser power and 10 s integration time. If required, the surface of the electrode was polarized at −0.9 V (vs. Ag/AgCl) during the SERS measurement.

### 2.6. DFT Calculations

The equilibrium geometries, normal mode vibrational frequencies, and the corresponding Raman intensities were obtained in the framework of the density functional theory (DFT). The ωB97X exchange-correlation functional was considered [26], combined with the D3-type empirical dispersion correction scheme [27,28], and applying the def2-TZVPP triple-zeta basis set of the Karlsruhe group [29], as implemented in the ORCA program suite [30,31]. The RIJCOSX approximation [32] designed to accelerate Hartree–Fock and hybrid DFT calculations were considered together with the Def2/J [33] auxiliary basis set for Coulomb fitting. In the case of silver and gold atoms, the def2-SVP(ECP) or the “split valence” double-zeta basis set together with the Dirac–Fock effective core potentials [34] was considered. Crystal structures, from which the silver and gold surfaces were built, were taken from the Crystallography Open Database (COD) [35] under the codes 9008459 and 9008463 [36].

### 2.7. Finite-Difference Time-Domain Electromagnetic Simulations

Finite-Difference Time-Domain simulations were performed using Ansys/Lumerical FDTD software. A three-dimensional simulation volume was considered, with dimensions X × Y × Z = 550 nm × 466 nm × 500 nm. The NP film was modeled using gold or silver spherical nanoparticles having diameters of 50 nm, arranged in a hexagonal lattice with a 2 nm interparticle spacing. The simulated configuration comprised two layers of NP, totaling 86 × 2 = 172 particles on a dielectric substrate. The so-called conformal variant 1 was used as a mesh refinement method, while boundary conditions included PML for all boundaries except the X direction for which the Anti-Symmetric feature was used. An additional mesh having a 0.4 nm step was used for the region with the metal particles. The “Johnson and Christy”/“CRC” data were used for gold/silver material properties. A plane wave source was propagated at normal incidence (along the Z axis) from the air side, above the nanoparticles. Reflectance spectra were obtained by placing a power monitor behind the source plane, while electric field maps were obtained from field profile monitors positioned perpendicular to the NP film in the XZ plane.

## 3. Results and Discussion

### 3.1. Morphology of the Self-Assembled Nanoparticle Films

**Figure 1:**
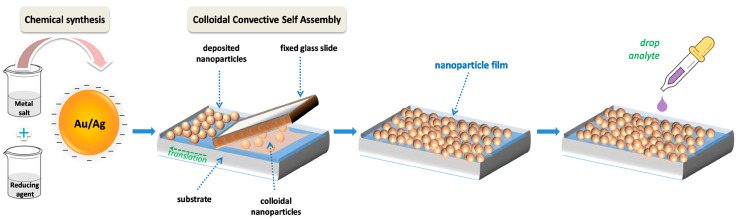
Schematic representation of the chemical synthesis and the convective self-assembly procedure.

The process employed for the fabrication of metal nanoparticle films is depicted in Figure 1 and it involves the main steps described in the following. Colloidal metal (Au, Ag) nanoparticles were first synthesized by chemical route; after being concentrated, the colloidal suspensions were deposited into films on solid substrates by convective self-assembly (CSA). The assembly process is based on the evaporation of water near the imposed linear triple-contact line, which induces the flow of particles from the suspension toward the meniscus region. In this way, by adjusting the speed at which the substrate is translated, dense, compact nanoparticle packing can be obtained on cm^2^ areas. These nanoparticle films were then used for SERS measurements of analytes deposited by simple drop-coating and drying.

**Figure 2:**
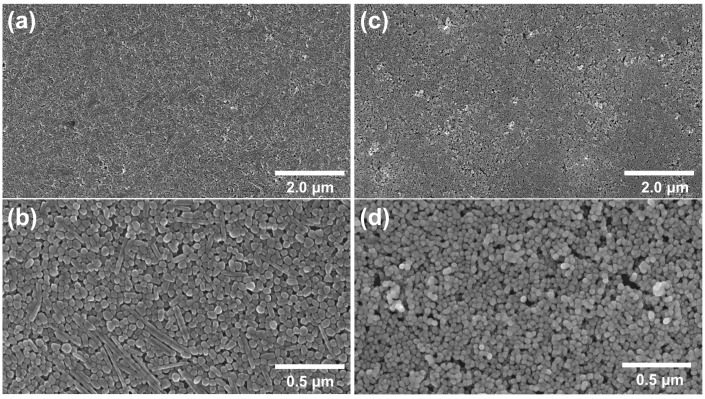
SEM images of (**a**,**b**) Ag NP film; (**c**,**d**) Au NP film.

For the fabrication of the NP films, the conditions of the chemical synthesis of Ag and Au colloids were adjusted such that nanoparticles of similar sizes would be obtained in both cases. Based on TEM image analyses (Figure S1), the obtained Ag NPs were in the size range of 40–60 nm, while the Au NPs were in the range of 50–65 nm. The morphology of the obtained films was investigated by SEM, with typical results presented in Figure 2. Both kinds of films exhibited a dense nanoparticle packing, as expected from the CSA method. The thickness of the nanoparticle films corresponded to a mixture of mono- and bi-layers.

### 3.2. SERS of Propranolol on Ag and Au NP Films

**Figure 3:**
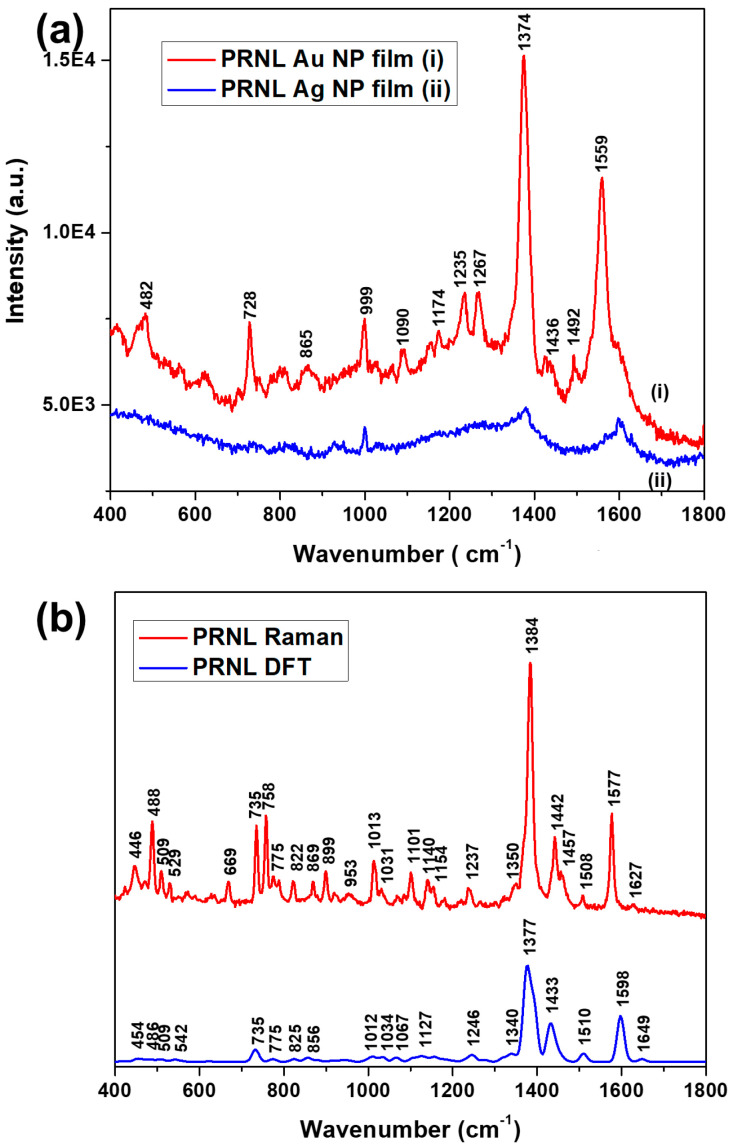
(**a**) SERS spectra of propranolol on Ag and Au NP films, respectively. (**b**) Experimental and calculated Raman spectra of propranolol.

SERS spectra obtained from PRNL adsorbed on silver and gold colloidal films, respectively, are presented in Figure 3a. The most striking observation is that the spectra of PRNL on Au NP film showed higher relative intensity compared to the ones obtained from the Ag NP film. The intensity of the 1374 cm^−1^ band was ~10 times higher on the Au NP film compared to the Ag one. In a previous work, we investigated in detail the SERS properties of the Ag NP films under multiple laser excitations and determined that the best enhancements are obtained for the 785 nm excitation, with the SERS enhancement factor reaching values larger than 10^5^ [37]. Notably, although nanoparticle sizes are slightly different for the Au and Ag NPs, they are similar enough not to induce considerable size-dependent SERS effects. The spectra of PRNL on Au NPs were dominated by the 1559, 1374, and 728 cm^−1^ bands. Other noticeable peaks were located at 482, 999, 1090, 1174, 1235, 1267, 1436, and 1492 cm^−1^. The SERS spectrum of PRNL acquired from the Ag NP film, on the other hand, presented only a few bands of weak intensity located at 1598, 1378, and 999 cm^−1^. One has to notice that the bands at 999 and 1602 cm^−1^ have been previously detected as a marker of the plastic substrate on which the metallic NPs are deposited, respectively, the carboxylate groups of adsorbed citrate ligands coordinated to the surface of the Ag NPs [37]. The relative intensity of the 999 cm^−1^ band in the SERS spectra obtained from PRNL on Au NP film, however, could also be an indicator for the presence of carbon rings in cyclic or aromatic compounds, responsible for the ring breathing mode of PRNL molecules. To conclude, the SERS spectra of PRNL acquired either from Ag NP or Au NP were dominated by the 1374 cm^−1^ band, assigned previously to ring breathing vibrations, as well as CH bending vibrations [11,38].

To acquire insight into the relationship between the molecular chemical structure and the Raman spectra, the calculated and experimental Raman spectra of propranolol are presented in Figure 3b. Selected experimental and simulated vibrational bands in the Raman spectrum of propranolol and optimized geometries of propranolol from DFT calculations are presented in Table S1 (Supplementary Material). The Raman spectrum of propranolol is dominated by the 1384 cm^−1^ (1377 cm^−1^ in the simulated spectrum) and 735 cm^−1^ bands assigned to the naphthalene ring vibrational modes. Other main Raman bands of PRNL are the CC stretching and CH bending vibrations of the naphthalene ring observed at 1577 cm^−1^ (1598 cm^−1^ in the simulated spectrum), the CH bending in the naphthalene ring at 1436 cm^−1^ (1433 cm^−1^ in the simulated spectrum), the 758 cm^−1^ assigned to NH, CN, and CC stretching vibrations, and the 488 cm^−1^ band corresponding to stretching vibrations of the naphthalene ring [13]. As a general observation, a good correlation was observed between the simulated Raman spectrum and the experimental one, from both the wavenumbers and relative peak intensities point of view, as can be seen in Figure 3 and in Table S1 (Supplementary Material).

When compared with the normal Raman spectrum of PRNL (Figure 3b), the SERS spectra showed modifications such as wavenumber shifts of at least 7 cm^−1^ for the most intense SERS modes, as well as enhanced intensities of PRNL characteristic Raman bands. These results point out the interaction between propranolol and the metallic surface. Previous research concluded that the disappearance or weak intensity of the C-O and C-N stretching vibrations from the SERS spectra, respectively, as well as the highest electron density located on the oxygen atoms, indicated that the PRNL adsorption to the Ag surface was performed mainly through a physisorption process involving the O atoms [13]. The orientation of the PRNL molecule relative to the metal surface was suggested based on the resulting molecular electrostatic potential (MEP) and the observed SERS bands, invoking the SERS selection rules. Since the SERS surface selections rules state that the normal modes with a change in polarizability component perpendicular to the surface are enhanced, it would appear that the naphthalene ring lies in a perpendicular orientation to the silver surface; thus, its π electrons cannot contribute to the adsorption ([12] and references therein).

In fact, the natural behavior of the PRNL molecule in its fundamental state is determined by the alkylic substituted -OR group directly bounded to the naphthalene ring in position 1 (as α type position), which exhibits both inductive electron-withdrawing effect (−I) as well as mesomeric electron-donating effect (+E) as an order I type substituent when preferentially activating the naphthalene ring in α position of the substituted aromatic ring. Through an extended *p*-π conjugation in the PRNL molecule, the delocalization of a lone pair (p electrons) of the -OR neutral oxygen atom bonded to the naphthalene aromatic ring in position α into the naphthalene aromatic ring (4 π electrons) ensures the formation of negative charge separated resonance form, localized in position 4 (as α type position) of the substituted naphthalene ring, as well as a resonance form with the negative charge delocalized between the oxygen atom and the carbon atoms of the substituted naphthalene aromatic ring. The more spread out the electrons are, the more stable the resonance form is. Therefore, a parallel arrangement of the naphthalene ring with respect to the metal surface is much more likely than a perpendicular or tilted one. This assumption is supported by the amplified intensity of the peaks assigned to the skeletal naphthalene ring vibrations and the CH stretching vibrations from the SERS spectra.

### 3.3. DFT Analysis

**Figure 4:**
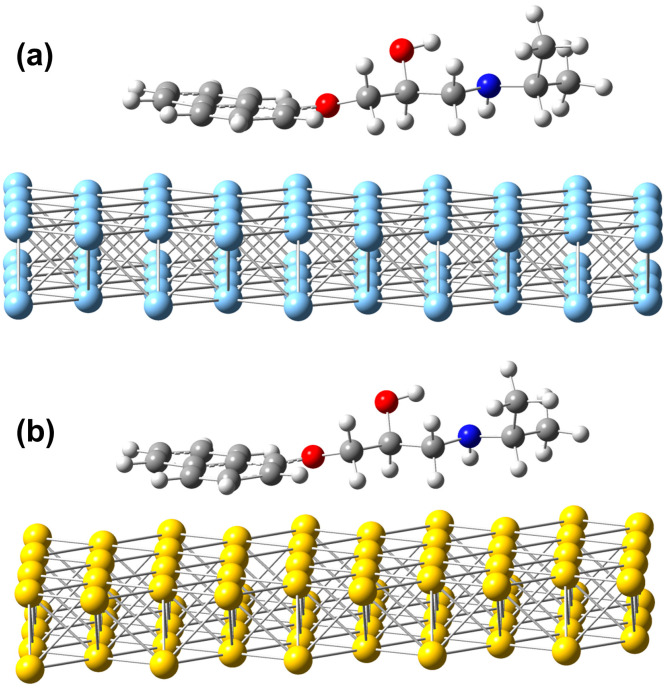
Equilibrium geometry structures of the propranolol adsorbed on the (**a**) silver and (**b**) gold surfaces.

DFT calculations were performed in order to investigate the interaction between PRNL molecules and silver or gold surfaces. Therefore, we performed a DFT analysis starting from a model of the molecule absorbed in a parallel orientation with respect to the metal surface. To take into account the electron distribution of metal surfaces, initially, a 4 × 4 × 5 crystal supercell was built, and its atomic charge population was computed considering the ωB97X-D3/def2-SVP(ECP) level of theory and summarizing the charge distribution for each layer built by the 4 × 5 supercell crystal sheets. The global picture of the charge distribution for the four crystal sheets shows an extra electron charge for the two inner sheets, while the outer surface of the crystal structure contains approximately one electron less charge. We then built a model of the molecule absorbed on the metal surface by taking a two-layer crystal sheet with the electron charge reduced by one and placing the propranolol molecule on it, as shown in Figure 4. 

The adsorbed energy considered as the intermolecular interaction energy between the propranolol and the metal surface was computed considering the ωB97X-D3 exchange-correlation functional, where the def2-TZVPP basis sets were used in the case of propranolol, while for the metal structure, the def2-SVP(ECP) basis sets were considered. The propranolol’s adsorption energy on the silver surface is −49.38 kcal/mol, while that of PRNL on the gold surface is −65.06 kcal/mol, demonstrating that adsorption of the PRNL on a silver surface is ~23% weaker than that on a gold surface. Since the dispersion interaction in the D3 approach is a post-energy correction, it is very easy to calculate the contribution of dispersion and non-dispersion interactions. Thus, for adsorption on the silver surface, the dispersion contribution is −36.03 kcal/mol and the non-dispersion energy component is −13.35 kcal/mol, while for the gold surface, these values are −43.46 kcal/mol and −21.59 kcal/mol, respectively, which leads to the conclusion that the dispersion interaction is very important for propranolol adsorption on both silver and gold metal surfaces. Moreover, if we perform the Löwdin population analysis, we observe that 0.45 e electrons of charge are transferred from the propranolol molecule to the silver surface, while the same charge transfer is only 0.37 e electrons for the gold surface. As for the deformation energy of propranolol on a metal surface, it is −1.35 kcal/mol for silver and −1.56 kcal/mol for gold. Since the metal surface–propranolol complex has a huge number of atoms, their Raman/SERS spectra are extremely difficult to compute. Accordingly, the theoretical normal mode analysis and the computation of the Raman spectra were performed only for the isolated propranolol molecule. Our present DFT calculations regarding the investigation of the interaction between propranolol and silver or gold surfaces are the first reported ones in the literature, to our knowledge. In summary, the DFT analysis suggests that the adsorption affinity of the propranolol molecule for the gold surface is higher than for the silver surface, proving a better interaction of this molecule with the Au surface. In this way, better resolved SERS spectra of propranolol on the gold surface are obtained, as compared with those corresponding to the silver surface.

### 3.4. Optical Response Analysis

**Figure 5:**
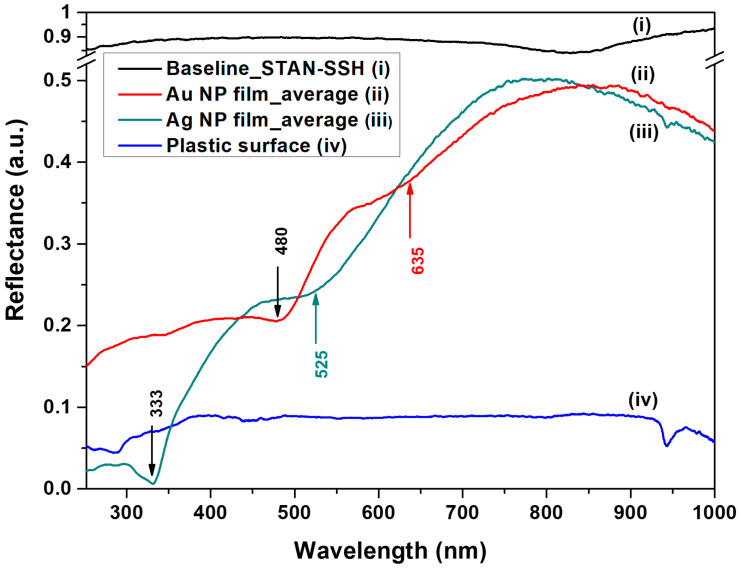
UV-VIS-NIR reflectance spectra of (i) an aluminum high-reflectivity specular reflectance standard; (ii) Au NPs film on plastic substrate; (iii) Ag NP film on plastic substrate; (iv) the plastic substrate.

In Figure 5, the UV-VIS-NIR reflectance spectra of the Ag NPs, as well as those of Au NPs films coated on a plastic substrate, in regard to a high-reflectivity Al mirror standard as reference, are presented.

The reflectance associated with localized surface plasmon resonance (LSPR) in noble metal nanoparticles [39] films assembled on the plastic substrate is noticed and analyzed. Thus, specific minima displayed by the spectral reflectance curves for flat Ag and Au metal mirrors at normal incidence [40] can also be observed in the case of Ag NPs film at 333 nm (0.4%) and for Au NPs film at 480 nm (21%), respectively. In the visible domain of the spectra, the response of both films showed a similar decrease in the reflectance of approximately 10% within a spectral region of around 150 nm. In the case of Ag NPs film, this region is situated between 600 nm and 455 nm, where the reflectance drops from 33% to 23%, displaying a minimum at 525 nm. The spectrum of Au NPs film displays this interval between 710 nm and 565 nm, where the reflectance drops from 44% to 34% and shows a minimum at 635 nm. The obvious diminished overall reflectance of both films in the visible spectral domain, where the Ag and Au NPs surface plasmon absorption prevails [41], indicates a nanostructured aspect of the surface rather than a flat one, with a mean size of particles around 50 nm [42]. It is worth mentioning that the position [43] and the intensity [44] of the plasmon absorption maxima of both films differ from those of the Ag and Au NPs colloids used in the convective assembly process due to the optical coupling between nanoparticles when in close proximity.

**Figure 6:**
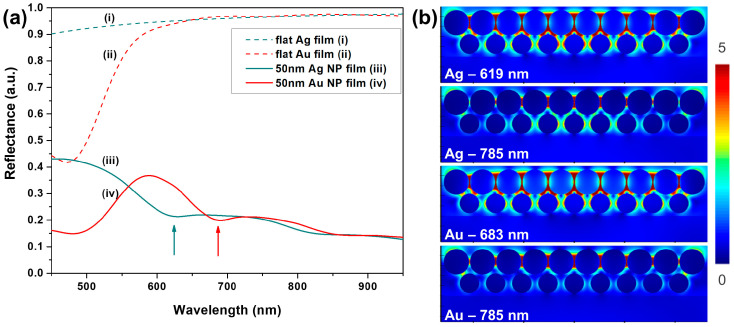
(**a**) Simulated reflectance spectra of: (i) flat Ag film; (ii) flat Au film; (iii,iv) double-layer Ag NP and Au NP films, respectively, made of 50 nm particles. (**b**) Electric field maps at 785 nm (laser excitation) and at the local reflectance minima indicated by arrows on panel (**a**).

FDTD simulations were also performed to help understand the behavior observed experimentally in the reflectance spectra. Note that the purpose of these simulations was not to reproduce the experimental results perfectly but mainly to assess the differences and similarities between the Ag NP and the Au NP films. To mimic as closely as possible the real NP films, we performed a full 3D simulation of the optical response of a finite NP array comprising a relatively large number of particles (172) disposed in two layers. Figure 6a presents the simulated reflectance spectra for the Ag NP and the Au NP arrays, together with the reflectance of flat Ag and Au films (100 nm thick), as references.

The main specific spectral features observed are the reflectance dips centered at 619 nm for the Ag NP array and 683 for the Au NP array, marked by arrows in Figure 6a. We attribute these dips to the interparticle plasmon coupling in the NP arrays, this being supported by the presence of the strongest electric fields at these wavelengths, as shown in Figure 6b. In addition to this mentioned dip, the NP films also exhibited a reminiscence of the behavior of flat films. This was shown by the minimum below 500 nm for Au, attributed to the onset of inter-band transitions of electrons in the metal. The overall behavior also matches rather well the experimental behavior discussed in the previous paragraph, with the observation that the red-shift of the dip position in the simulation relative to the experiment can be attributed to the obvious higher degree of ordering and uniformity in the modeled structure. By further analyzing the electric field in the NP arrays at 785 nm, the laser wavelength used for SERS measurements, one can observe that the electric field distributions were very similar for the Ag NP and Au NP arrays. Moreover, we also analyzed the electric field spectrum at the gap between two NPs at the central position of the array (Figure S2 in the Supplementary Material). It showed that the electric field takes very similar values for the two metals, with a minor advantage for the Au NP array, in the range 785–881 nm (corresponding to 0–1400 cm^−1^ in the SERS spectra). To conclude this section, these simulations indicate a similar SERS performance of the Ag NP and Au NP arrays from the point of view of the achievable electromagnetic enhancements.

### 3.5. SERS Detection on Au NP Films

**Figure 7:**
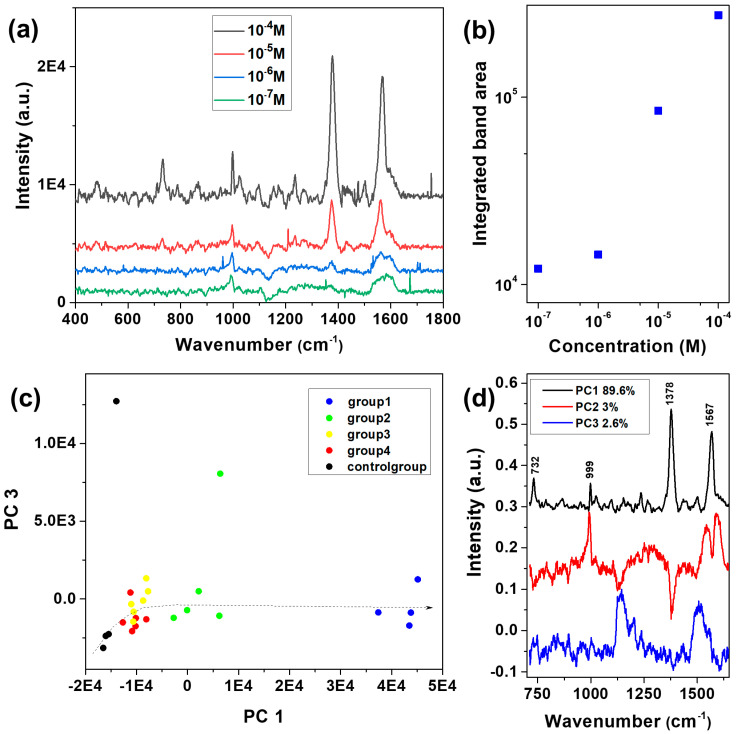
(**a**) SERS spectra of propranolol at different concentrations from 10^−4^ M to 10^−7^ M, obtained on Au NP film detection platform; (**b**) Integrated area of the SERS band at 1377 cm^−1^, as a function of propranolol concentration; (**c**) Scatter plot of PC1 vs. PC3 scores showing the differentiation of concentration-dependent SERS groups (10^−4^–10^−7^M), including the control group (bare Au NP film); (**d**) The loadings of the first three principal components indicating the SERS peaks that contribute most to the clustering between the five groups of spectra.

Despite the similar electromagnetic enhancements of Au and Ag NP films, respectively, indicated by the FDTD simulations, the comparatively performed SERS experiments, as well as the DFT investigations, indicated improved SERS detection of PRNL adsorbed on the Au NP film. Therefore, further experiments were performed on the Au NP film. Limit of detection analyses were carried out by testing different propranolol concentrations and monitoring the characteristic SERS markers of this drug. We obtained a general trend of decreasing the recorded intensities upon lowering the concentration of PRNL solution from 10^−4^ M to 10^−7^ M, as observed in Figure 7a. Moreover, the integrated area of the characteristic SERS mode at 1377 cm^−1^, which comes from naphthalene ring vibrations, decreased upon lowering the PRNL concentration down to 10^−7^ M (Figure 7b). Notably, the detection of PRNL at 10^−7^ M concentration, corresponding to 26 ppb, could be directly achieved using a simple analyte drop-coating protocol.

Further, we showed that PCA could be used to differentiate between the SERS spectra of PRNL even at low concentrations where the intensity of the characteristic PRNL SERS bands are reduced considerably to be easily identified by the naked eye. The data set was comprised of five groups of SERS spectra, representing different concentrations of PRNL in the 10^−4^–10^−7^ M, including the control group, which consisted of the spectra acquired from the pure Au NP film. Figure 7c presents the clustering of the five groups of spectra using the first and the third principal components (PCs). Together, these two PCs accounted for approximately 93% of the total variance in the data set. The loadings of PC1 (Figure 7d) presented the main SERS bands of PRNL and represented the major contributor of the first three PCs used for differentiating the concentration-dependent SERS groups. Group 1, which contained the spectra acquired from the highest PRNL concentration (10^−4^ M), was arranged on the far-right side of the scatter plot at high positive values of PC1. Group 2, comprised of slightly less intense SERS spectra of PRNL at 10^−5^ M, was gathered around low positive values of PC1, with some spectra falling on the negative side as well. Groups 3 and 4, representing the SERS spectra acquired from PRNL at 10^−6^ M and 10^−7^ M, respectively, were entwined around similar negative values, however, separated along PC3, while the control group was clustered at the far negative side of PC1. The loadings of PC1, which accounted for 89.6% of the total variance, captured significant features corresponding to PRNL, such as the peaks located at 1567, 1496, 1446, 1378, 1268, 1234, and 732 cm^−1^, which were observed in the SERS spectra characteristic to PRNL adsorbed on the Au NP film, as well. The second PC presented, in addition to two of the main bands of propranolol (1377 and 1567 cm^−1^), the contribution of the SERS substrate observed at 993 and 1020 cm^−1^.

### 3.6. Au NP Films as Electrodes for EC-SERS Assays

**Figure 8:**
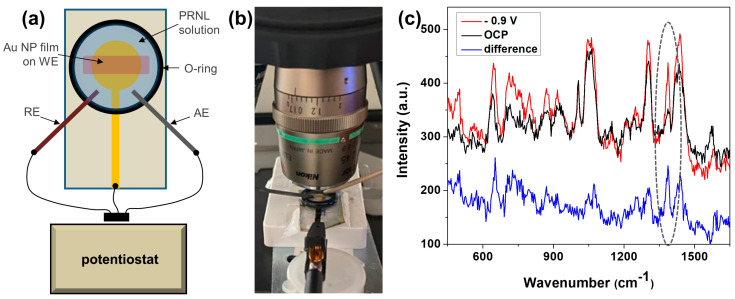
(**a**) Scheme and (**b**) picture of the EC-SERS setup. (**c**) EC-SERS spectrum of PRNL at −0.9 V in PBS (pH 7), SERS spectrum at OCP, and their difference.

The possibility of using these self-assembled NP films as working electrodes for EC-SERS studies was further investigated. Successful experiments may bring several advantages for analytical applications, such as improvements in detection sensitivity and/or reproducibility [6]. Polarizing the surface of the electrode during a SERS measurement can impact both the chemical and the electromagnetic enhancement mechanisms [45]. The Au NP film was employed here as a roughened electrode, and the EC-SERS was performed in water. To assess the optimal polarization potential for PRNL detection, reference EC-SERS measurements were performed using electrochemically-roughened screen-printed gold electrodes and PRNL solution in PBS (10^−5^ M). The surface of the electrode was polarized from negative to positive values, and the acquired spectra are shown in Figure S3. Further, EC-SERS measurements were performed using the Au NP film as working electrode. The potential-dependent SERS spectra of PRNL revealed an optimum polarization potential of −0.9 V (vs. Ag/AgCl), offering the highest SERS signal enhancement. On the other hand, at positive potentials, the obtained signal was mitigated (Figure S3). The recorded SERS spectra of PRNL on self-assembled NP films at open circuit potential (OCP) and −0.9 V are presented in Figure 8. PRNL SERS bands were observed at 1572 cm^−1^ (weak intensity), 1437 cm^−1^ (medium intensity), 1387 cm^−1^ (strong intensity), 916 cm^−1^ (weak intensity), 869 cm^−1^ (medium intensity), 797 cm^−1^ (medium intensity), 710 cm^−1^ (medium intensity), 645 cm^−1^ (medium intensity), and 496 cm^−1^ (medium intensity). It is interesting to notice that the enhanced vibrational modes were not necessarily the same as the ones observed in the SERS experiments. Additionally, the SERS bands detected in both experiments appear, usually, red-shifted in the EC-SERS data. These observations point to modifications in the electronic distribution of PRNL molecules induced by polarization. 

The SERS and EC-SERS results presented above are competitive when compared with previous results in the literature. Even though PRNL has been previously studied with SERS [3,4,6,7], experimental data in these works were not collected on self-assembled colloidal nanoparticle films but on colloidal suspensions. The authors, Levene et al., performed SERS measurements on aggregated Ag and Au colloids, respectively. It can be clearly observed from Figure S5 of this reference [10] that in the absence of an aggregating agent, the SERS spectra show irreproducible results. In our paper, we showed that PRNL can be detected label-free at low concentrations without using aggregating agents and that the spectra were reproducible regardless of the PRNL concentration. Moreover, SERS spectra for propranolol on both silver and gold nanoparticles are presented in only one reference [6]. It is also worth mentioning that the LoD of 2.36 ng/mL (7.97 nM) propranolol reported in ref. [4] is a predicted value and not a measured one for detecting this β-blocker. The authors developed a multiobjective evolutionary algorithm (MOEA) based on Pareto optimality to improve enhancement and reproducibility in SERS. In this MOEA approach, a combination of five different colloids with six different aggregating agents was tested, and a wide range of concentrations for both were explored [4]. Other authors detected PRNL down to concentrations of 10^−5^ M in body fluids [46]. The LOD of 5 × 10^−7^ M was achieved based on calculations on the linear regression curve and represents an estimation. On the contrary, the results presented in our paper were obtained experimentally by measuring PRNL at the 10^−7^ M concentration deposited on the Au NP film. Additionally, it is worth mentioning again that, in our case, no aggregating agents are needed for PRNL detection. Moreover, one of the main aims of the study was to determine the plasmonic metal with the highest SERS efficiency for PRNL detection when used in a configuration of self-assembled colloidal nanoparticle films.

The EC-SERS results point toward the successful implementation of the Au NP film as working electrodes for EC-SERS experiments. Although the results are preliminary and analyses need to be continued, they demonstrate the feasibility of using the Au NP films as platforms for EC-SERS studies. To the best of our knowledge, this is the first reported detection of propranolol in an EC-SERS experiment, and at the same time, electrodes for EC-SERS assays made of colloidal nanoparticles deposited into films on solid support by CSA were not previously reported. Further, by using EC-SERS, the sensitivity could be even further increased, and such substrates could be used for the future detection of PRNL from surface waters at relevant concentrations. It is also interesting to note that gold surfaces are preferred over silver for many EC-SERS applications due to their wider hydrogen overpotential and, thus, a wider range of operational potentials and higher overall chemical and electrochemical inertness. Finally, as demonstrated by previous works, such self-assembled nanoparticle films could also be used for electrical resistance-based chemical sensing [47] or deformation sensing [48], pointing toward interesting possibilities for developing multi-stimuli-responsive sensing devices.

## 4. Conclusions

Ag and Au colloidal nanoparticles were assembled into high-density films on solid substrates by convective self-assembly. SERS of propranolol on these plasmonic platforms was explored, and Au NP films yielded more intense and well-resolved SERS spectra than Ag NP films. Based on theoretical DFT analysis, it was found that propranolol’s adsorption energy on the silver surface is −49.38 kcal/mol, while that on the gold surface is −65.06 kcal/mol, demonstrating that the interaction between propranolol and silver surface is weaker by about 23% compared with the interaction with the gold surface. The optical reflectance spectra supported by FDTD electromagnetic simulations also showed that Au NP films present a plasmonic response that is better adapted to SERS enhancements using the 785 nm laser excitation compared to the Ag NP films. Therefore, the stronger SERS signal of PRNL on Au NP films can be attributed to the synergistic effect of its better interaction with the metal surface and the slightly higher electromagnetic field enhancements provided by the Au surface. Detection of propranolol at 10^−7^ M concentration, corresponding to 26 ppb, was demonstrated to be achievable by a straightforward drop-coating protocol.

Future research directions include lowering the LoD of PRNL, as well as detecting it in real water samples collected from contaminated areas. In order to decrease the LoD, SERS substrates exhibiting higher EFs need to be designed. Moreover, functionalization of the surface of the metallic NPs could help improve both LoD and specificity in detecting PRNL, even in complex matrices. Regarding the EC-SERS measurements, further optimization of the metal-based electrodes can be achieved, which can lead to a decrease in the achieved LoD, as well.

Finally, constructed SERS-active working electrodes made of Au NP films over flat Au electrodes proved to be promising platforms for developing various sensitive and/or selective EC-SERS applications. These results contribute to a better understanding of SERS of propranolol on noble metal solid SERS substrates, point to the right metal choice for the development of SERS-based propranolol detection protocols and also highlight a novel approach for a simple realization of nanoparticle-based EC-SERS platforms.

## Data Availability

The data presented in this study are available on request from the corresponding author.

## Supplementary Materials

The following supporting information can be downloaded at: https://www.mdpi.com/article/10.3390/bios13050530/s1. Figure S1: TEM images of Ag NPs and Au NPs; Figure S2: Electric field magnitude at the central position of the simulated NP array, between two NPs, for both Ag (blue) and Au (red). The inset is a zoom on the area of interest 785–881 nm, corresponding to 0–1400 cm^−1^ in the SERS spectra; Figure S3: Potential dependent SERS spectra of 10^−4^ M PRNL in PBS (pH 7) from OCP to −1.1 V in 100 mV increments (left) and SERS spectra at selected potentials (right); Table S1: Selected experimental and simulated vibrational bands in the Raman spectrum of propranolol. Optimized geometry of propranolol from DFT calculations is presented.
