# Supplementary material for: Gold vs. Silver Colloidal Nanoparticle Films for Optimized SERS Detection of Propranolol and Electrochemical-SERS Analyses
Source: Biosensors (Basel). 2023 May 9;13(5):530. doi: 10.3390/bios13050530 (PMC10216585; doi:10.3390/bios13050530)
Supplement: Supplementary file 1 [file biosensors-13-00530-s001.zip › biosensors-2366104-supplementary.pdf]

biosensors

MDPI

Supplementary Materials

Gold vs Silver Colloidal Nanoparticle Films for Optimized SERS Detection of Propranolol and Electrochemical-SERS Analyses

Cristina M. Muntean[1&], Denisa Cuibus[1&], Sanda Boca[2,1], Alexandra Fălămaş[1], Nicoleta Toşa[1], Ioana Brezeştean[1], Attila Bende[1], Lucian Barbu-Tudoran[1], Rebeca Moldovan[3], Ede Bodoki[3], Cosmin Farcău[1,2*]

[1]National Institute for Research and Development of Isotopic and Molecular Technologies, 67-103 Donat Str., 400293 Cluj-Napoca, Romania
[2]Institute for Interdisciplinary Research in Bio-Nano-Sciences, Babeş-Bolyai University, 42 T. Laurian Str., 400271 Cluj-Napoca, Romania
[3]Analytical Chemistry Department, Faculty of Pharmacy, „Iuliu Hațieganu" University of Medicine and Pharmacy, 4, Louis Pasteur, 400349 Cluj-Napoca, Romania

[&] C.M.M. and D.C. contributed equally to this work.

[*]Corresponding author:
Dr. Cosmin Farcău, email: cfarcau@itim-cj.ro

Table S1. Selected experimental and simulated vibrational bands in the Raman spectrum of propranolol. Optimized geometry of propranolol from the DFT calculations is presented.

Wavenumber (cm^{-1})		Vibrational assignment [1,2]
**Theoretical**	**Experimental**	
486 (vw)	488 (s)	Symmetric longitudinal stretching of the naphthalene ring 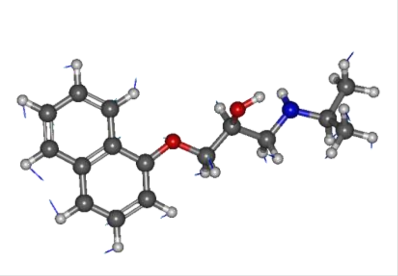
509 (vw)	509 (w)	Asymmetric longitudinal stretching of the naphthalene ring
	669 (w)	γ(CC) + γ(CH)
735 (w)	735 (s)	Naphtalene ring breathing 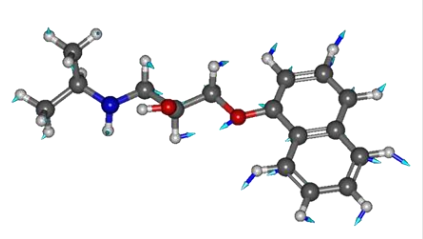
	758 (s)	γ(NH) + υ(CN) + tυ(CC)
775 (vw)	775 (w)	γ(CH) + γ(CC) (naphthalene) 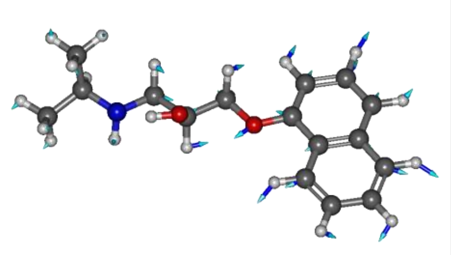
825 (vw)	822 (w)	δ(CCC)
856 (vw)	869 (w)	δ(CCC) + δ(COC) 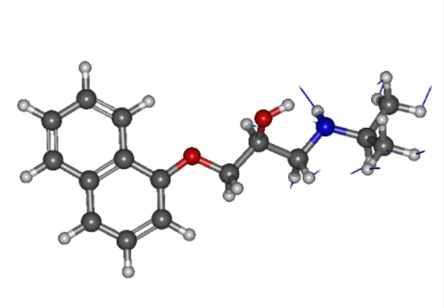
	899 (m)	δ(CH) + υ(CO) + δ(NH)

		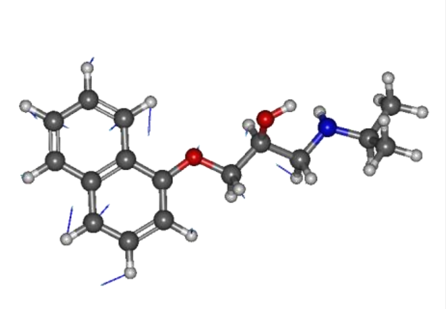
1012 (w)	1013 (m)	υ(CC) + δ(CH)
	1101 (m)	υ(CO) + tδ(CH) + δ(CCC) 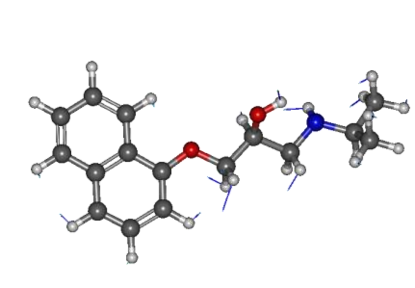
1127 (w)	1140 (m)	δ(CH) 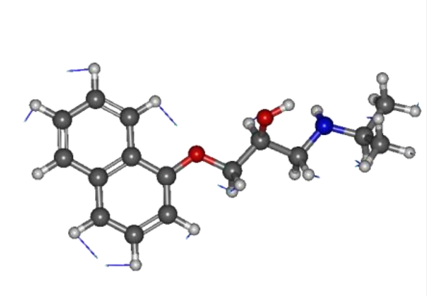
1246 (w)	1237 (w)	δ(CH) + δ(OH)
1377 (vs)	1384 (vs)	υ(CC) (naphthalene) + δ(CH) 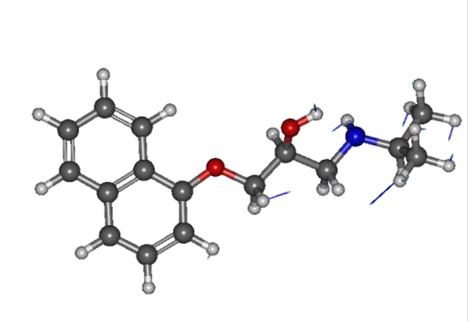
1433 (m)	1442 (m)	δ(CH) (naphthalene) 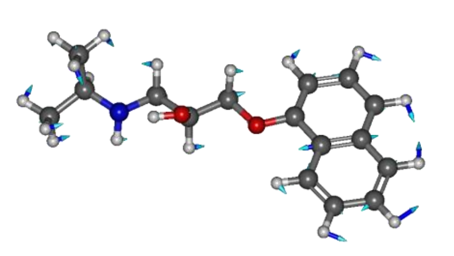

1510 (w)	1508 (w)	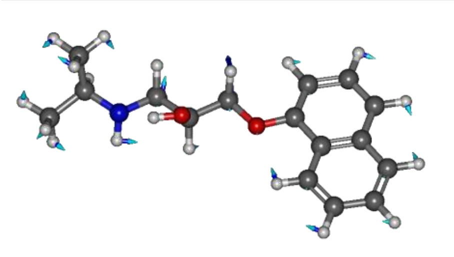
1598 (s)	1577 (s)	υ(CC) + δ(CH) (naphthalene) 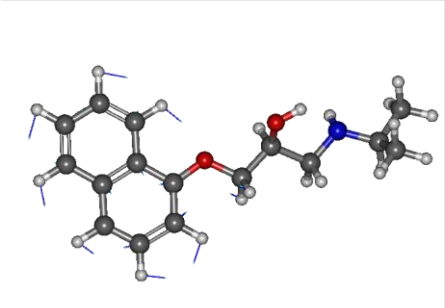

Abbreviations: vs—very strong; s—strong; m-medium; w—weak; vw—very weak; Γ—out-of-plane bending; υ—stretching; δ—bending.

Transmission electron microscopy (TEM) images.

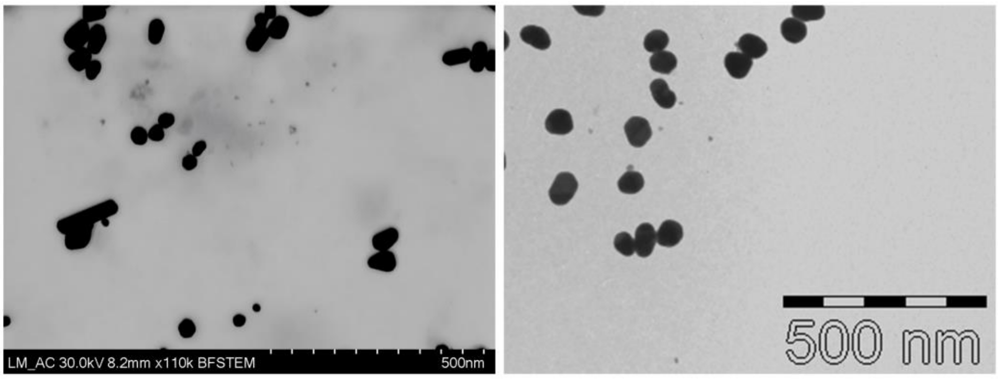

Figure S1. TEM images of Ag NPs (left) and Au NPs (right).

Electric fields in the NP arrays.

The electric field spectrum at the gap between the two NPs at the central position of the array was analyzed for both the Ag and the Au NP arrays. The magnitude of the electric fields is presented in Figure S1. Despite a marked difference for the maximum of each system, at the wavelengths of interest, under 785 nm excitation, the difference between Ag and Au is less pronounced.

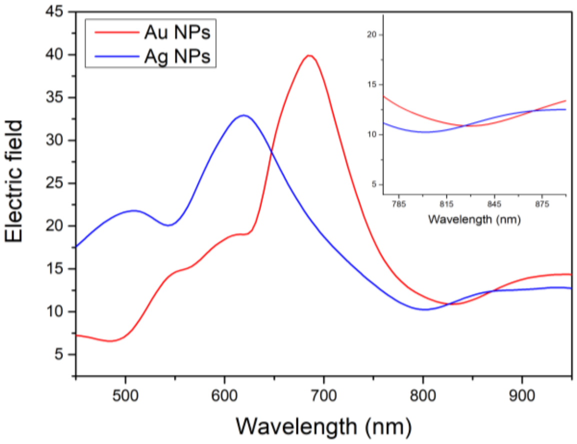

Figure S2. Electric field magnitude at the central position of the simulated NP array between two NPs, for both Ag (blue) and Au (red). The inset is a zoom of the area of interest 785–881 nm, corresponding to 0–1400 cm^{-1} in the SERS spectra.

Potential-dependent SERS spectra of propranolol.

The potential-dependent SERS spectra of PRNL was recorded on electrochemically roughened gold-based screen-printed electrodes (DRP250BT, Metrohm DropSens, Spain), with the same instrumentation used for EC–SERS measurements (Section 2.5). No additional electrodes were needed, as the reference (Ag) and auxiliary electrodes (Pt) were co-imprinted on the ceramic support. As a study sample, a 10^{-4} M PRNL solution in PBS was used. The parameters for SERS measurements were 5 s integration time, two averages and 3.05 mW laser power. The surface of the electrode was polarized from OCP to -1.1 V in 100 mV increments. At the end of the experiment, the influence of a positive potential (+0.5V vs. Ag) was also tested.

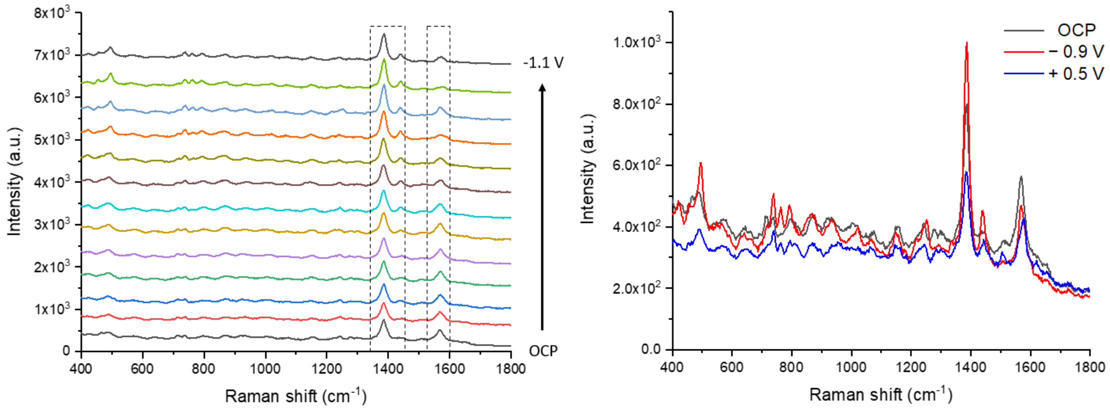

Figure S3. Potential-dependent SERS spectra of 10^{-4} M PRNL in PBS (pH 7) from OCP to -1.1 V in 100 mV increments (left), and SERS spectra at selected potentials (right).

References

[1] R. Stiufiuc, C. Iacovita, C.M. Lucaciu, G. Stiufiuc, R. Nicoara, M. Oltean, V. Chis, E. Bodoki, Adsorption geometry of propranolol enantiomers on silver nanoparticles, Journal of Molecular Structure. 1031 (2013) 201–206.

[2] A. Farcaș, C. Iacoviță, E. Vințeler, V. Chiș, R. Știufiuc, C.M. Lucaciu, The Influence of Molecular Structure Modifications on Vibrational Properties of Some Beta Blockers: A Combined Raman and DFT Study, Journal of Spectroscopy. 2016 (2016) 3137140. https://doi.org/10.1155/2016/3137140.
